# Immune-related biomarkers in triple-negative breast cancer

**DOI:** 10.1007/s12282-021-01247-8

**Published:** 2021-04-09

**Authors:** Juan Zhang, Qi Tian, Mi Zhang, Hui Wang, Lei Wu, Jin Yang

**Affiliations:** Department of Medical Oncology, The First Affiliated Hospital of Xi’an Jiaotong University, No. 277 West Yanta Road of Xi’an, Xi’an, 710061 People’s Republic of China

**Keywords:** TNBC, Heterogeneity, Immune-related biomarker, Immunotherapy, Prognosis

## Abstract

Breast cancer is a commonly diagnosed female cancer in the world. Triple-negative breast cancer (TNBC) is the most dangerous and biologically aggressive subtype in breast cancer which has a high mortality, high rates of relapse and poor prognosis, representing approximately 15–20% of breast cancers. TNBC has unique and special biological molecular characteristics and higher immunogenicity than other breast cancer types. On the basis of molecular features, TNBC is divided into different subtypes and gets various treatments. Especially, immunotherapy becomes a promising and effective treatment to TNBC. However, not all of the TNBC patients are sensitive to immunotherapy, the need of selecting the patients suitable for immunotherapy is imperative. In this review, we discussed recent discoveries about the immune-related factors of TNBC, including tumor-infiltrating lymphocytes (TILs), programmed death-ligand protein-1 (PD-L1), immune gene signatures, some other emerging biomarkers for immunotherapy effectivity and promising biomarkers for immunotherapy resistance. In addition, we summarized the features of these biomarkers contributing to predict the prognosis and effect of immunotherapy. We hope we can provide some helps or evidences to clinical immunotherapy and combined treatment for TNBC patients.

## Introduction

Breast cancer is known as the most common cancer and becomes one of the main death reasons in female [1]. It is classified into different subtypes according to the expression of molecular features, which includes Luminal A, Luminal B, HER-2 overexpression and triple-negative breast cancer (TNBC). TNBC is the most aggressive cluster of all breast cancers with a rapid progression, high probabilities of early recurrence, and distant metastasis, making up about 15–20% of breast cancers [2]. The primary and traditional established approaches to treat TNBC patients are surgery, radiation and chemotherapy with serious toxic and side effects. What’s more, those patients with a poor response to neoadjuvant treatment and no pathologic complete response (pCR) especially show a poor prognosis and a high risk of distant relapse, typically within the first 2–3 years after initial diagnosis. Therefore, the need of novel and better treatment options is dramatically emerging.

Massively parallel sequencing and other ‘omics’ technologies have revealed unexpected heterogeneity of TNBC [3]. The understanding of the immune profiles with features of TNBC is clearer than before [4]. TNBC has unique biological molecular characteristics and the immunogenicity is higher than other breast cancer subtypes. Immunotherapeutic approaches have shown great and promising developments in recent studies of breast cancer [5], and several clinical trials have shown that immunotherapy could improve clinical outcomes and prognosis of TNBC patients. For example, in Impassion 130 clinical trial, the patients whose tumors have PD-L1 expression level ≥ 1% with unresectable locally advanced or metastatic TNBC received atezolizumab plus nab-paclitaxel have a prolonged progression-free survival (PFS) and overall survival (OS) [6]. The immunotherapy becomes a new and effective treatment option to TNBC, but not all of the TNBC patients are sensitive to immunotherapy.

To maximize the benefits and minimize the toxicities of cancer immunotherapy, we need to discriminate the patients who will benefit from and get a better response to immunotherapy from others. The immune-related biomarkers become necessary [7]. The high expression level of tumor-infiltrating lymphocytes (TILs), programmed death-ligand protein-1 (PD-L1), highly tumor mutational burden (TMB), microsatellite instability (MSI), and mis-match repair deficiency (MMR) are the features of TNBC [8], which may contribute to TNBC patients suitable and sensitive to immunotherapy. These characteristics could be considered as predictors for the efficacy of immunotherapy in breast cancer [9]. From a meta-analysis, PD-L1-positive, first-line therapy, non-liver metastasis, high TILs and CD8 + T-cell infiltrating levels could predict a better response to immune checkpoint inhibitor (ICI) treatment [10]. However, the practical accuracy and usefulness of immune-related biomarkers which could predict promising therapeutic outcomes in TNBC are still controversial. In this review, we summarized the recent progresses and discoveries about the immune-related factors of TNBC. And we analyzed these novel and appropriate immune-related biomarkers which may contribute to predict the response and efficiency of immunotherapy or resistance to immunotherapy.

## Existing and under study immune-related biomarkers

### Tumor-infiltering lymphocytes (TILs) in TNBC

The vast heterogeneity of TNBC mainly originate from the tumor immune microenvironment, which is associated with tumor cell proliferation and aggressive ability, metastasis and drug resistance [11]. TILs comprising different levels of lymphocyte and monocyte infiltration is the main component in tumor immune microenvironment [3], which can be evaluated in H&E pathologic sections on the basis of established guidelines [12]. Currently, various evidences in scientific interest and clinical setting both have shown that the density, type, and location of TILs in TNBC exhibit different values for assessing disease prognosis and progression.

First of all, in the quantitative level, X. Yu et al. showed that higher value of total tumor-infiltrating lymphocytes (both intraepithelial and stromal) counts associated with better prognosis (pooled HR 0.88, 95% CI 0.83–0.94) in TNBC than other breast cancer subtypes in a 17 eligible studies including 12,968 candidates. Whether disease-free survival (DFS) or metastasis-free survival, which is statistically significant (*P* < 0.0001). A long overall survival also was indicated by total TILs, but without statistically significant (*P* = 0.08) [13]. Loi et al. demonstrated that high levels of TILs could significantly predict the rates of distant recurrence, and that each 10% increase of TILs was associated with a 13% decrease in relative risk of distant recurrence in a study with 134 TNBC patients [14]. TILs also can be viewed as a predictive factor of response to chemotherapy. For TNBC patients who received neoadjuvant chemotherapy, high expression levels of immune-related markers or immune infiltration were correlated with higher pCR, lower risk of relapses and better outcomes. Denkert, C., et al. indicated that the percentage of intratumoral lymphocytes was a significant independent parameter for pCR in both cohorts (training cohort: *P* = 0.012; validation cohort: *P* = 0.001). And the pCR rates of 42% (training cohort) and 40% (validation cohort) in lymphocyte-predominant breast cancer (LPBC) were higher than those tumors without any infiltrating lymphocytes with pCR rates of 3% (training cohort) and 7% (validation cohort) [15]. In two national randomized clinical trials (ECOG 2197 and ECOG 1199) with 481 evaluable TNBC patients had TILs (sTILs, 80%; iTILs, 15%) using contemporary adjuvant chemotherapy, Adams, S., et al. observed that higher sTIL scores were associated with better prognosis. With the increase of sTILs, the risk of recurrence or death reduce. They also confirmed that sTILs should be as a robust prognostic factor in TNBC [16]. Some other clinical trials which could show the correlation between TILs and outcomes in TNBC immunotherapy which is positive (Table1). In KEYNOTE-086 (a phase II trial), among the patients who received pembrolizumab (PD-1 inhibitor) with high stromal TILs levels had an improved ORR in metastatic TNBC. It means that TILs is emerging as a potentially important biomarker to predict the response of immunotherapy in TNBC [17].These findings and trials provide a strong support for the patients with immunotherapy in TNBC [4].

 Secondly, certain subgroups of TILs which contain different cellular composition of the immune infiltration represent different immune response and outcomes. We can regard it as qualitative differences. Various lymphocytes components of TILs induce different types of tumor microenvironment. Diverse forms affect the balance of immune response and escape, leading to different prognosis and outcomes in cancers [13]. On one hand, it can induce immunity, killing tumor cells, inhibiting tumor proliferation and progress. The majority of TILs are prominent CD8 + cytotoxic T lymphocytes (CTLs), which are the major effector cell type in breast cancer and related to a better prognosis [18]. And other immune cells contribute to a protective effect and favorable outcomes, such as CD4 + T helper, natural killer (NK) cells, M1 macrophages, and dendritic cells (DCs) [19]. On the other hand, the immune system can be suppressed by regulatory T cells, which further form the immune microenvironment to help the survival of tumor cells and promote carcinogenesis. The higher enrichment scores of macrophages M2, immature DCs, and eosinophils are suggested for a worse OS [20]. Foxp3 + T and PD-1 + T cells infiltration in tumor cells mediate tumor immune escape and might be a worse prognosis predictor in breast cancer [13, 21]. A high ratio of CTLs to FOXP3 + T cells plays a key regulatory role in the development and function of T regulatory cells, which is a strong predictor of pCR in the neoadjuvant setting [22]. Thirdly, the location of TILs also needs to be considered seriously. Significantly different from other solid tumors, stromal TILs (sTILs) shows a more important and meaningful prognostic value than CTLs (iTILs) in breast cancer [16, 22]. But X. Yu et al. thought that TILs location did not affect the prognosis of breast cancer patients, both iTILs and sTILs contributed to a favorable survival time [13]. In addition, the immunity decreased after tumor metastasis. It means the metastatic lesions had lower TILs and PD-L1 expression levels than primary tumor sites [23]. Perhaps this conclusion could hint at why early immunotherapy is more effective to patients. All in all, TILs have been recognized as the most valuable predictive biomarker, but the accuracy still needs to be confirmed [24]. Whatever, based on previous clinical trials and researches, we can regard it as an important factor related to prognosis.

**Table1:** The correlation between TILs and outcome in TNBC immunotherapy trials

Study	Line	Phase	Regimen	Method/content	Sample size	Correlation with outcome
*Early TNBC*						
KEYNOTE-173 [25]	Ib	Untreated, unresectable locally advanced TNBC	Pembrolizumab	Hematoxylin–eosin-stained sections (H&E) / sTILs	60	Higher quantities of pretreatment sTILs and PD-L1 CPS and on-treatment sTILs were significantly associated with higher pCR rates and ORR in primary TNBC treated with pembro and neoadjuvant treatment (NAT)
*Advanced TNBC*						
KEYNOTE-086 [26]	III	Advanced, untreated, any PD-L1 TNBC (cohort A) Advanced, untreated PD-L1 + TNBC (cohort B)	Pembrolizumab	Hematoxylin–eosin-stained sections (H&E) /sTILs	Cohort A: 147 Cohort B: 46	Higher TILs levels were associated with significantly improved ORR (odds ratio 1.26, 95% CI 1.03–1.55, *P* = 0.01) and DCR (odds ratio 1.22, 95% CI 1.02–1.46, *P* = 0.01). PD-L1 expression significantly correlated with TILs levels (*ρ* = 0.4962, *P* < 0.001)
KEYNOTE‐119 [27, 28]	III	mTNBC (metastatic TNBC) unselected for PD-L1	Pembrolizumab	Hematoxylin–eosin-stained sections (H&E) /TILs	622	Better OS for TILs ≥ 5% (0.75[0.59–0.96]) in the pembrolizumab arm but not the chemotherapy arm (1.46[1.11–1.92]). And TILs levels as a continuous variable were significantly (P < 0.05) associated with all clinical outcomes
Impassion130 [29, 30]	III	Untreated mTNBC unselected for PD-L1	Atezolizumab	Hematoxylin–eosin-stained sections (H&E) /sTILs	902 (451 treated with atezolizumab)	Patients whose tumors contained sTILs and expressed PD-L1 on immune cells had a significant improvement in PFS (HR 0.53; 95% CI, 0.38–0.74; *P* ≤ .005) and OS (HR 0.57; 95% CI, 0.35–0.92; *P* = 0.02)
TONIC [31]	II	Previously treated and untreated mTNBC	Nivolumab	Hematoxylin–eosin-stained sections (H&E) /sTILs	67	Significantly higher levels of sTILs and higher levels of CD8 and PD-L1 on immune cells in responders than in non-responders

### PD-L1 expression in TNBC

Programmed cell death receptor ligand 1 gene (PD-L1, also known as CD274 or B7-H1) significantly binds the PD-1 pathway and negatively regulates effector T-cell function [32]. Mittendorf et al. found the expression of PD-L1 is higher in TNBC than other breast cancer subtypes according to The Cancer Genome Atlas (TCGA) RNA sequencing data [33]. A meta-analysis collecting six studies which included 7877 cases has showed that a high expression level of PD‐L1 might lead to the mitigation of the host's anti-tumor immune response by activating of the immune checkpoint PD-1/PD‐L1 pathway, thus resulted in increased tumor aggressiveness [34] including inhibiting T-cell proliferation, promoting immune cell apoptosis, and might help these tumors evade anti-cancer immune responses.

The expression of PD-L1 in different locations induces different outcomes. If PD-L1 is expressed on cancer cells, it means this tumor is more malignant and aggressive, prone to metastasis and have a worse prognosis. A research published in 2014 has shown that high expression of PD-L1 on tumor cells could reflect the immune microenvironment which was associated with an adaptive immune resistance [35]. On the contrary, PD-L1 expressed on TILs showed low-risk clinicopathological parameters and a durable survival time in breast cancer [36]. Recently, Sugie T., et al. reported that PD-L1 expression on tumor cells and on immune cells is significantly correlated with TILs levels and infiltration of CD8 + T cells in TNBC using multiplex fluorescent IHC, which indicated that high level of PD-L1 on IC might reflect T cell-inflamed tumors with the amount of TILs present, including the CD8 + T cells required for anti-tumor responses [37]. Yuan et al. observed metastatic lymph nodes had higher expression of PD-L1 compared to primary tumor, in a study with 47 paired breast tumor and metastatic axillary lymph node samples [38].

Clinical trials targeting the PD-1/PD-L1 pathway to treat TNBC patients are ongoing, and preliminary results have been promising. The result is a bit different between early TNBC and advanced TNBC with immunotherapy. In early TNBC, patients with PD-L1 positive can get a higher pCR, but no statistically significant difference. With the increase of PD-L1 expression, the pCR also increases and survival time gets prolonged. Advanced TNBC patients with PD-L1 positive have a better outcome and longer survival time than PD-L1 negative patients. Keynote-522 is a phase 3 trial which added pembrolizumab to neoadjuvant chemotherapy in early TNBC. The conclusion is among all 602 patients, the people who accepted pembrolizumab–chemotherapy obtained a significantly higher percentage of pCR at the time of definitive surgery than people in the placebo–chemotherapy group. No matter PD-L1 is expressed or not, the combination therapy group always get a longer survival time. But the PD-L1-positive group had a higher pCR [39].

From JAVELIN solid tumor study, a phase 1 study, the metastatic TNBC patients treated with avelumab had higher ORR with a higher expression level of PD-L1 [40]. Keynote-119 showed that the therapeutic effect of pembrolizumab is enhanced with the increase of combined positive score (CPS) [41]. From the Impassion130 biomarker subgroup analysis, expression of PD-L1 on immune cells was required for response to the combination of atezolizumab plus nab-paclitaxel. In this clinical trial, the patients were evaluated by the expression of PD-L1 on immune cells using SP142 antibody, Ventana. In the ITT analysis, the mPFS was 7.2 months with atezolizumab plus nab-paclitaxel, as compared with 5.5 months with placebo plus nab-paclitaxel. Among patients with PD-L1-positive tumors, the mPFS was 7.5 and 5.0 months, respectively. In the ITT analysis, the median OS was 21.3 months with atezolizumab plus nab-paclitaxel and 17.6 months with placebo plus nab-paclitaxel. Among patients with PD-L1-positive tumors, the median OS was 25.0 and 15.5 months, respectively. It is evident that PFS and OS are longer, as well as the efficiency had been proven in PD-L1 + IC patients (the threshold is PD-L1 > 1%) [42]. However, the results of the final analysis of IMpassion130 in ESMO2020 mentioned that while OS differences for atezolizumab + nab-paclitaxel vs placebo + nab-paclitaxel in the IMpassion130 ITT population were not statistically significant, precluding formal testing, clinically meaningful OS benefit was observed in PD-L1 + IC patients (7.5-mo median OS improvement) [43].

Some other trails with immunotherapy also provided evidences about the prognostic and predictive value of PD-L1 expression in breast cancer. We listed them in Table 2. In SABCS2018, a scientifically complex study aimed at determining which patients will have the highest response rates to an immunotherapy agent known as atezolizumab, the same agent in the IMPASSION study. That trial showed patients with a marker called PD-L1 benefit from treatment with atezolizumab. In March 2019, atezolizumab plus nab-paclitaxel was approved could be used to treat PD-L1-positive metastatic, locally advanced, or unresectable TNBC by the FDA, based on the data from the Impassion130 trial. In addition, the Ventana PD-L1 (SP142) assay was also requested as a companion diagnostic device to select TNBC patients for atezolizumab [44]. 

In short, testing the expression of PD-L1 is of great significance in unresectable locally advanced or metastatic TNBC who might choose immunotherapy. But the criterion of PD-L1 expression level test is still in the dispute. PD-L1 expression is evaluated by using different pathological factors, antibodies, and cut-off points, which may cause various results in different institutions and platforms. The standardized method has not been established and confirmed by a consensus conference in the world. Now in clinical therapy or clinical trials, to test the expression of PD-L1, PD-L1 22C3 (Agilent Technologies Inc., Santa Clara, CA, USA), 28-8 (Agilent Technologies Inc.), SP142 (Roche Tissue Diagnostics, Tucson, AZ, USA), SP263 (Roche Tissue Diagnostics), and 73-10 (Agilent Technologies Inc.) have been taken into consideration [45]. Rugo HS et al. presented a report at ESMO 2019. They tested the PD-L1 status in available samples from IMpassion130 by using VENTANA SP142 or SP263 IHC assay (IC ≥ 1%, SP142 + or SP263 +) or Dako PD-L1 IHC 22C3 assay (CPS ≥ 1, 22C3 +). And they found PD-L1 + prevalence was 46% for SP142 + , 81% for 22C3 + , and 75% for SP263 + . More patients with PD-L1 + tumors were identified by using 22C3 and SP263 assays at the evaluated cutoffs. But the patients with PD-L1 + expression tested by SP142 obtained the greatest clinical benefit with atezolizumab plus nab-paclitaxel, which proved that assessment with SP142 helps to extract the most effective population for treatment [46]. Emens LA et al. published a paper to discuss the differences in the evaluation methods of PD-L1 in SP142 staining which assessed PD-L1 IC and TC status. Scoring was based on PD-L1-expressing IC as a percentage of tumor area and PD-L1 scoring on TC was based on the percentage of PD-L1-expressing TC. Threshold of both is 1%. They found PD-L1 IC and TC were weakly correlated as continuous variables (r = 0.26). PD-L1 TC + prevalence was low, but most PD-L1 TC + samples were also PD-L1 IC + . Their analyses on IMpassion130 patients showed that PD-L1 on TC per se is not associated with the clinical activity of atezolizumab plus nab-paclitaxel. So SP142 assay with testing PD-L1 IC status should be as a clinically validated companion diagnostic for patients with newly diagnosed metastatic TNBC to decide if they could benefit from first-line treatment with atezolizumab plus nab-paclitaxel [30]. In addition, Winer EP et al. explained the advantages of CPS which was tested by 22C3 over other testing methods at SABCS 2020. PD-L1 expression in tumor and immune cells both are important in metastatic TNBC which could be as a predictive biomarker of pembrolizumab efficacy in KEYNOTE-119 cases [47]. So based on the researches published, we prefer the two approaches, SP142 by Ventana and 22C3 by Dako Agilent, which have been approved by FDA [32].

**Table2:** The correlation between PD-L1 and outcome in TNBC immunotherapy trials

Study	Line	Phase	Regimen	Antibody/cut-off value	Sample size	Correlation with outcome
*Early TNBC*						
KEYNOTE-522 [39]	III	Locally advanced TNBC unselected for PD-L1	Pembrolizumab	Agilent PD-L1 IHC 22C3 pharmDx / CPS:1	1174	For pembrolizumab vs placebo, pCR (ypT0/Tis ypN0) was 68.9 vs 54.9% in the PD-L1 + population and 45.3 vs 30.3% in the PD-L1-population. The addition of pembrolizumab to chemo followed by pembrolizumab showed a favorable trend in EFS (HR 0.63 [95% CI, 0.43–0.93])
KEYNOTE-173 [25]	Ib	Untreated, unresectable locally advanced TNBC	Pembrolizumab	Agilent PD-L1 IHC 22C3 pharmDx / CPS:1	60	A high pretreatment PD-L1 combined positive score and high counts of TILs were significantly associated with higher pCR rates
NeoTRIP [48, 49]	III	Early high-risk or locally advanced unilateral TNBC	Atezolizumab	VENTANA PD-L1 IHC SP142 / IC:1%	180	pCR is higher in atezolizumab group (43.5 vs 40.8%). And 51.9% in PD-L1( +) group. The presence of PD-L1 expression was the most significant factor influencing rate of pCR (OR 2.08). Whatever, all results were not statistically significant
Impassion031 [50]	III	Untreated stage II–III histologically documented TNBC	Atezolizumab	VENTANA PD-L1 IHC SP142/IC:1%	333	pCR is 58% in the atezolizumab plus chemotherapy group and 41% in the placebo plus chemotherapy group (rate difference 17%, 95% CI 6–27; one-sided *p* = 0·0044 [significance boundary 0·0184]). In the PD-L1-positive population, pCR is 69% (95% CI, 0.57–0.79) with 53 of 77 patients in the atezolizumab plus chemotherapy group versus 49% (95% CI, 0.38–0.61) with 37 of 75 patients in the placebo plus chemotherapy group (rate difference 20%, 95% CI, 0.04–0.35; one-sided *p* = 0·021 [significance boundary 0·0184]). Early-stage TNBC can benefit from atezolizumab regardless of PD-L1 status
*Advanced TNBC*						
Impassion130 [29]	III	Untreated mTNBC unselected for PD-L1	Atezolizumab	VENTANA PD-L1 IHC SP142 / IC:1%	902 (451 treated with atezolizumab)	The ORR is 56% (58.9% in PD-L1 +) with mOS 25 months(atezolizumab) vs mOS 18 months(placebo) in PD-L1 +
NCT01375842 [51]	Ib	Advanced TNBC unselected for PD-L1	Atezolizumab	VENTANA PD-L1 IHC SP142 / IC:1%	116	The ORR is 10% in total (12.7% in PD-L1 +), with mPFS 1.4 months And the ORR first line is 24%, ≥ 2 lines is 6%
KEYNOTE-012 [52]	Ib	Advanced TNBC unselected for PD-L1	Pembrolizumab	Agilent PD-L1 IHC 22C3 pharmDx / CPS:1	111	65 (58.6%) had PD-L1–positive tumors with ORR 18.5%, DOR 2.1 months, mPFS 1.9 months, mOS 11.2 months
KEYNOTE-086 [17, 53]	III	Advanced, untreated, any PD-L1 TNBC (cohort A) Advanced, untreated PD-L1 + TNBC (cohort B)	Pembrolizumab	Agilent PD-L1 IHC 22C3 pharmDx/CPS:1	Cohort A: 170 Cohort B: 84	Cohort A: The ORR is 5.3% in total, 5.7% in PD-L1 positive and 5.3% in PD-L1 negative, respectively. With mPFS 2 months Cohort B: The ORR is 21.4% and the mPFS is 2.1 months
KEYNOTE-150 [54]	Ib/II	Advanced TNBC unselected for PD-L1	Pembrolizumab	Agilent PD-L1 IHC 22C3 pharmDx / CPS:1	107	The ORR is 26.4% (30.6% in PD-L1 +) with mPFS 4.2 months, mOS 17.7 months
KEYNOTE‐119 [27, 28]	III	mTNBC unselected for PD-L1	Pembrolizumab	Agilent PD-L1 IHC 22C3 pharmDx / CPS:1	622	No improved outcome in ITT population or PD-L1 + tumors. PD-L1 with higher expression lever, pembrolizumab is more effective. Among ITT group with CPS > = 1, CPS > = 10, CPS > = 20, OS HR: 0.96,0.86,0.78,0.58; PFS HR: 1.60,1.35,1.14,0.76; ORR: 9.6 vs10.6%, 12.3 vs9.4%, 17.7%vs9.2%, 26.3 vs11.5%
KEYNOTE-355 [55]	III	Untreated TNBC	Pembrolizumab	Agilent PD-L1 IHC 22C3 pharmDx / CPS:1 and 10	847	PFS is longer in PD-L1-positive (CPS > = 10) group, the mPFS increase by 4.1 months. (HR: 0.65; *P* = 0.0012). A trend towards improved efficacy with PD-L1 enrichment was observed in patients treated with pembrolizumab + chemotherapy
TONIC [31]	II	Metastatic or incurable locally advanced TNBC	Nivolumab	Agilent PD-L1 IHC 22C3 pharmDx / PD-L1 in tumor cells (TC): 1 and 5%; PD-L1 in immune cells (IC): 1 and 5%	67	Higher TILs (median 12.5% versus 6%, p = 0.004) and PD-L1 on IC (median 15 vs 5%) on responders versus non-responders. Better PFS and OS was observed in PD-L1 IC ≥ 5% patients. No difference was observed between PD-L1 TC ≥ 1 and < 1% populations
JAVELIN [40]	Ib	Locally advanced or mTNBC unselected for PD-L1	Avelumab	Dako PD-L1 IHC 73–10 pharmDx / PD-L1 in tumor cells: 1, 5 and 25%; PD-L1 in tumor-associated immune cells: 10%	58	The confirmed ORR was 5.2% in patients with TNBC, with higher ORR in PD-L1 + tumor-associated immune cells versus PD-L1- tumor-associated immune cells TNBC (22.2 vs 2.6%)

TNBC triple-negative breast cancer, PD-L1 programed death-ligand 1, mPFS median progression-free survival, mOS median overall survival, ORR overall response rate, pCR pathological complete response, OR odds ratios, HR hazard ratio.

### Immune gene signatures in TNBC

Different immune cell types express different levels of immune gene signatures with various functions and immune effect mechanisms associated with clinical benefits in TNBC [3].The study collecting 193 TNBC patients have found an inverse association between immune metagene expression and clonal heterogeneity, somatic copy number alteration levels. Lymphocyte-rich TNBCs with better prognosis had significantly lower mutation than lymphocyte-poor TNBCs, which means immune-rich TNBCs were associated with lower clonal heterogeneity. The results were also established in TCGA data set and METABRIC data set [56]. Therefore, it is necessary to develop immune gene signatures into the prognostic and predictive biomarkers in the future treatment for breast cancer [57].

As we know, some factors can be tested from gene levels to evaluate the potential clinical benefits of immunotherapy in cancer. The most multigene assays that have been confirmed were immune checkpoint genes (CTLA-4, IDO1, LAG3, PDCD1, PDL1 and so on) associated to efficiency of ICIs [58]. The expression of PD-L1 is higher in TNBC which contributes to effective responding to immunotherapy as we explained in the last part. Clinically meaningful OS benefit was observed in PD-L1 + patients from the final analysis of IMpassion130 in ESMO2020 [43].

From previous clinical trials and researches, TMB has also been listed to be a predictive biomarker of response to ICIs treatments in different types of cancers [59]. The patients with high TMB were more sensitive and responsive to immunotherapy. But the role of TMB in TNBC remains controversial. Because contrary to other tumors, mutational load is relatively low in breast cancer, suggesting that TMB is unable to contribute to identifying patients benefit from immunotherapy [60]. The present study showed that TMB was not strongly associated with cytolytic activity and different degrees of immune cell infiltration in the immune microenvironment of TNBC which owned the highest expression of immunoregulatory molecules than other kinds of breast cancer [61]. In Impassion130 study, TMB was not regarded as a predictive biomarker of immunotherapy in TNBC. But in KEYNOTE-119 from 2020 ASCO, it was reported that the ORR has been improved in patients with TMB ≥ 10 mut/Mb, which was 14.3% in pembrolizumab group vs. 8.3% in chemotherapy group, compared with 12.7% in pembrolizumab group vs. 12.8% in chemotherapy group with TMB < 10 mut/Mb. The OS also got prolonged in TMB ≥ 10 mut/Mb population [62].

At the same time, Kraya, A.A., et al. calculated median HRD-total scores which were determined for BRCA1/2, homologous recombination (HR) mutant, and HR wild-type tumors. Then used the scores as the basis for dichotomization for each group of tumors, like homologous recombination repair (HRD)-low group and HRD-high group. They demonstrated that HRD-low TNBC tumors were the most immunogenic subset with high PD1/PD-L1 and TCR signaling, while HRD-high tumors were the least immunogenic in breast cancer [63].

DNA mis-match repair (MMR) system is a type of gene surrogates in tumors. The main reason for tumor is the MMR defect, which leads to the accumulation of genetic sequences of errors, commonly referred to as microsatellites, and presents a microsatellite instability-high (MSI-H) phenotype. MSI-H and mis-match repair deficiency (dMMR) are arguably predictive biomarkers for clinical response to ICIs in solid tumors [64]. Recently, the patients with dMMR or MSI-H across five clinical trials treated with pembrolizumab (KEYNOTE-016, 164, 012, 028, 158) showed a durable response in colorectal, endometrial, biliary, gastric, esophageal, pancreatic and breast cancers [65]. The patients with dMMR could get a longer PFS and OS in colorectal cancer. As well as the responses were durable [66].

Some other new immune gene signatures caught more attentions in recent years. Bernards R, et al. found N-MYC-mediated down-modulation of MHC class I antigen expression [67]. Layer JP, et al. reported a T-cell-poor microenvironment is associated with genomic amplification of the MYCN (N-MYC) proto-oncogene in primary metastatic neuroblastomas [68]. In SABCS2020, Lee JV, et al. reported MYC overexpression is loss of MHC class I in breast cancer, which is related to tumor immune. Tokumaru, Y., et al. established that patients with enrichment of KRAS signaling gene sets were associated with inflammation and favorable tumor immune microenvironment and the patients with KRAS-high owned significantly better DFS and OS than KRAS-low patients in TNBC [69]. Cheng, J.N., et al. demonstrated that TP53 and PIK3CA might be feasible biomarkers to select patients who would benefit most from ICIs by analyzing TCGA database. The patients with the TP53MutPIK3CAWild genotype might improve the response to immunotherapy, which might contribute to precise immunotherapy in TNBC [61].

### Other biomarkers for breast cancer immunotherapy

Because tumor tissue biopsy is not easy to get regularly, circulating ‘liquid biopsy’ biomarkers have been noticed as predictive and prognostic factors which can be non-invasively obtained from patients and trended over time. There are a lot of liquid biomarkers found in predicting the response to immunotherapy. The most biomarkers we know are circulating tumor cells (CTC), cell-free DNA (cfDNA) or circulating tumor DNA (ctDNA) [70], as well as exosomes. Some new blood index are under study, LDH, neutrophil–lymphocyte ratio (NLR), absolute eosinophil count, monocyte count and myeloid-derived suppressor cells (MDSCs), T-cell markers and soluble PD-L1 (sPD-L1), B cell-antibody markers, soluble CD25 (sCD25), blood tumor mutational burden (bTMB) and so on. But all of them were confirmed in non-small cell lung carcinoma (NSCLC) or melanoma or oral squamous cell cancer (OSCC) which are still not clear in breast cancer immunotherapy [44]. A relationship between the microbiome and cancer is an ongoing area of research. Gut microbiota is related to the local and systemic innate and adaptive immune responses which may lead to chronic inflammation and cancer. Routy et al*.* found the outcome of PD-1 blockade could be significantly influenced by gut microbiome in mice and patients. And antibiotic consumption was associated with poor response to immunotherapeutic PD-1 blockade in lung and kidney cancers [71]. In 112 metastatic melanoma patients starting treatment with anti–PD-1 therapy, Gopalakrishnan, V., et al. indicated that the gut microbiome might modulate responses to anti-PD-1 immunotherapy. The patients with a favorable gut microbiome enhanced systemic and anti-tumor immune responses by increasing antigen presentation and improving effector T-cell function in the periphery and the tumor microenvironment [72]. But we do not have an accurate definition and conclusion about the effect of gut microbiome to immunotherapy. Still we should believe that there must be some connections between them.

### Biomarkers for immunotherapy resistance

Not all of the patients respond to immunotherapy. Non-responders are resistant to immunotherapy when they start to receive treatment called primary resistance. And others generated acquired resistance during therapy or after relapse. Sharma, P., et al. divided resistance into primary and adaptive resistance and explained the mechanisms from tumor cell intrinsic factors and extrinsic factors separately. In the aspect of tumor cell intrinsic factors, the mechanism include absence of antigenic proteins (low mutational burden, lack of viral antigens, lack of cancer-testis antigens, overlapping surface proteins), absence of antigen presentation (deletion in TAP, deletion in B2M, silenced HLA), genetic T-cell exclusion (MAPK oncogenic signaling, stabilized β-catenin, mesenchymal transcriptome, oncogenic PD-L1 expression) and insensibility to T cells (mutations in interferon gamma, pathway signaling). To tumor cell extrinsic factors, absence of T cells (lack of T cells with tumor, antigen-specific TCRs), inhibitory immune checkpoints (VISTA, LAG-3, TIM-3) and immunosuppressive cells (TAMs, Tregs) were listed [73]. The suppression of anti-tumor immune responses and the progression of cancer were mainly influenced by the increase in the recruitment and activation of immunosuppressive cells, such as Tregs, Bregs, TAMs, and MDSCs within the TME. In addition, the cross-talk between non-immune cells, for example CAFs and TECs, also contributed to the resistance of immunotherapy [74]. Most of these ultimately are driven by any number of developments: tumor mutations and adaptations, reduced neoantigen generation or expression, disfunction of MHCs, resistance to IFN-γ signaling, indoleamine 2,3-dioxygenase (IDO) overexpression, loss of phosphatase and tensin homologue (PTEN) expression, and overexpression of the Wnt–β-catenin pathway and so on [75, 76].

Based on the mechanisms, potential biomarkers are related to immunogenic antigen landscape and preexisting immune context [77]. PD-L1 was regarded as a predictive biomarker for immunotherapy. However, PD-L1-positive patients are not always responsive to immunotherapy and PD-L1-negative people may benefit from it. We cannot just define PD-L1 must be a positive biomarker for immunotherapy. Form another dimension to, it is likely that PD-L1 is in connection with immunotherapy resistance [66]. Because PD-L1 expression can be induced by IFN-γ, which is related to an active anti-tumor immune response, it was referred to as a mechanism of adaptive immune resistance [73].

Janus kinases (JAK) have been reported related to resistance to immunotherapy. In a mechanistic study, Sucker et al. indicated mutations in JAK2 could increase IFN-γ resistance and reduce subsequent anti-PD-1 therapy failure in melanoma patients [78]. Zaretsky, J.M., et al. analyzed the whole-exome sequencing data based on matched baseline and recurrent biopsy samples from four metastatic melanoma patients who received anti-PD-1 (Pembrolizumab) and experienced initial objective regression of the tumor followed by progression months to years later. They proved immunotherapy resistance-related functional deletion mutation in the gene encoding interferon-receptor-associated JAK1 or JAK2, along with a wild-type allele. Truncation of JAK1 and JAK2 mutations leads to a lack of response to interferon, including insensitivity to its antiproliferative effects on cancer cells [79].

Mutations in beta-2-microglobulin (B2M) disrupt antigen presentation, leading to immune checkpoint blockade therapy resistance [77]. An increase in B2M mutations was significantly related to an increase in PD-1 + T-cell infiltration, indicating that drug resistance caused by B2M mutation is associated with PD-1 + T-cell infiltration [80]. Ishizuka, J.J., et al. showed that loss of function of ADAR1 restores sensitivity to immunotherapy in tumors with a B2M deletion by inactivating antigen presentation by tumor cells [81].

In melanoma, PTEN deletion promotes AKT phosphorylation, thereby promoting PI3K/AKT pathway activation, and ultimately promotes PD-L1 expression, thereby inactivating T cells. In addition, PTEN inhibits the expression of immunosuppressive factors IL-10, IL-16, and VEGF which may contribute to immunotherapy resistance. The activation of Wnt–β-catenin signaling pathway was associated with loss of T‐cell gene expression characteristics in melanoma. And in colorectal cancer (CRC) tumors can also significantly reduce the infiltration of CD8 + T cells, which might result in resistance to immunotherapy [82]. Additionally, the secretion of inhibitory molecules IDO can have a direct negative effect on T-cell function in the microenvironment [83]. And the combination of IDO inhibitors and immunotherapy has been shown to increase TILs and their functional capacities in the TME.

**Fig. 1:**
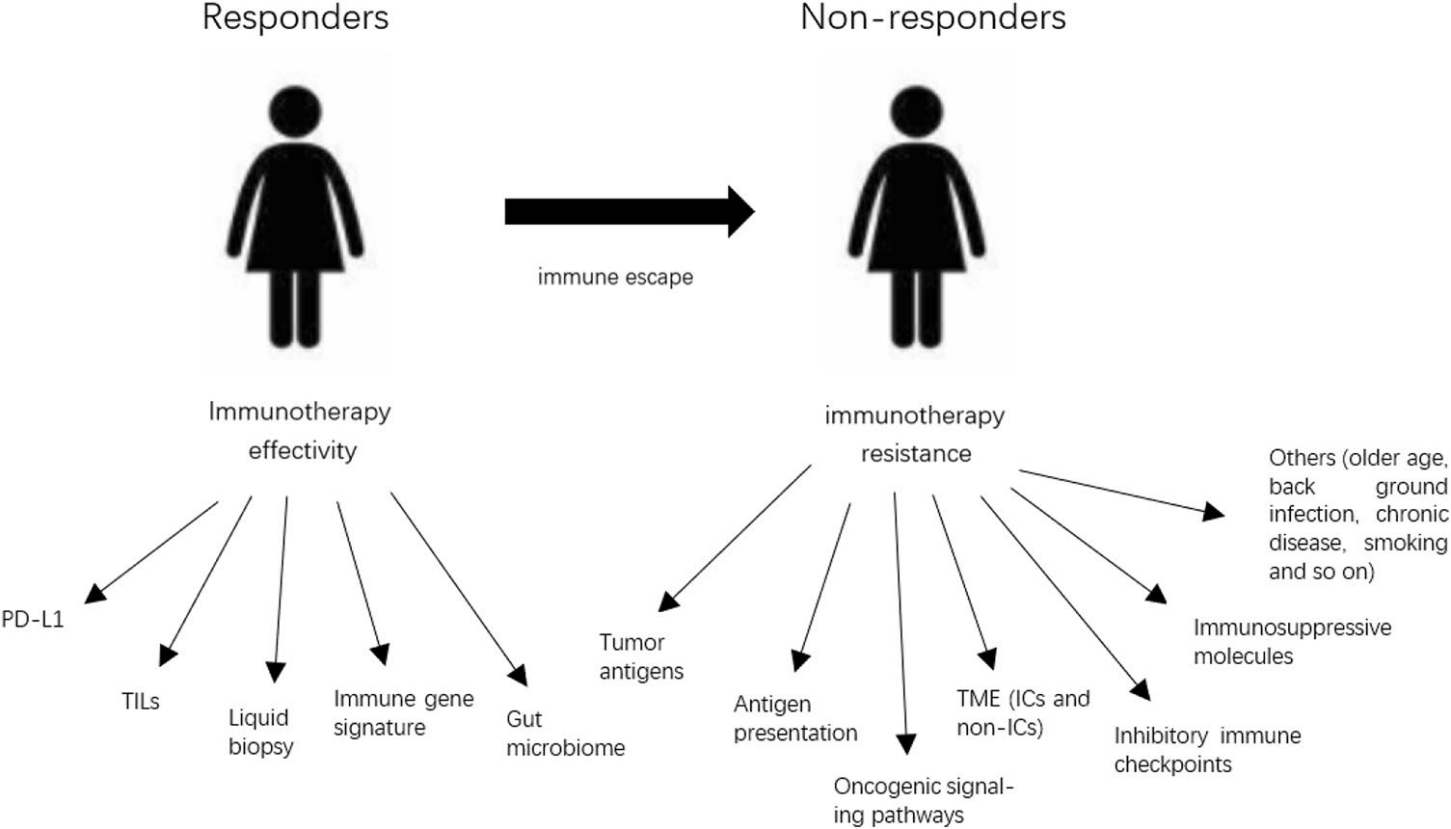
The potential biomarkers and mechanisms for clinical response or resistance to immunotherapy. (1) PD-L1, programmed cell death receptor ligand 1; (2) TME, tumor microenvironment; (3) ICs, immune cells, including TAMs (tumor-associated macrophages), Tregs (T regulatory cells), MDSCs (myeloid-derived suppressor cells); non-ICs, non-immune cells, including CAFs (cancer-associated fibroblasts), TECs (tumor endothelial cells)

There are also some other factors which can increase chance for resistance to immune-checkpoint blockers (ICB) immunotherapy, including older age, background infection or chronic disease, smoking, gut microbiota and so on [84]. In conclusion, the mechanisms of resistance to immunotherapy are still under research. But we still can find some way to stimulate immune functions or change the TME to improve the efficiency of immunotherapy, including combined treatment targeting immune and non-immune targets, chemotherapy, or radiotherapy (Fig. 1).

## Results and conclusion

Immunotherapy is a promising and effective treatment approach to TNBC. Whatever, some patients still cannot respond to immunotherapy. Therefore, it is necessary to identify immune-related biomarkers which can figure out these patients and define risk stratification to achieve accurate treatment. In this review, we listed different immune-related biomarkers in TNBC, including TILs, PD-L1, immune gene signatures, and other biomarkers, which may be helpful and valuable. Moreover, we explained the promising biomarkers for immunotherapy resistance. As reported, including tumor antigens, TME, signaling pathway, immune molecules and so on also could affect the responses to immunotherapy [45]. TNBC has a high degree of TILs which contribute to a lower risk of disease relapse and a better prognosis than those with a low level of TILs.

At the same time, the treatment of ICIs in metastatic TNBC (mTNBC) with high TILs shows promising results, indicating the potential benefits of immunotherapy for these patients with TNBC [3]. From a research published in 2019, TILs and immune checkpoint molecules were suggested as potential biomarkers to predict the therapeutic efficacy of selected ICIs in TNBC with "immune-inflamed” cluster by Xiao, Y., et al. [85]. The meaning of PD-L1 is still ambiguous because no consistent evaluation standard. Immune gene signatures and other new biomarkers like liquid biopsy and gut microbiome are also still being researched. In addition, the clinical benefits of TNBC patients were improved by the understanding of the impact of different drug components [11]. It is likely that we can use composite biomarkers other than single biomarker to predict clinical outcomes in response of immunotherapy or combined treatment [45]. So developing advanced methods or using multiple biomarkers to make joint prediction is the focus of future investigation. And in our opinion, we prefer to use PD-L1 and TILs as a more comprehensive composite biomarker. We hope this review can help to recognize the immune-related biomarkers as the important factors to predict response and prognosis for immunotherapy and combined treatment in TNBC.
